# Using peer workers with lived experience to support the treatment of borderline personality disorder: a qualitative study of consumer, carer and clinician perspectives

**DOI:** 10.1186/s40479-020-00135-5

**Published:** 2020-09-02

**Authors:** Karlen R. Barr, Michelle L. Townsend, Brin F. S. Grenyer

**Affiliations:** School of Psychology and Illawarra Health and Medical Research Institute, University of Wollongong, Wollongong, NSW Australia

**Keywords:** Borderline personality disorder, Peer support, Consumer peer worker, Carer peer worker, Recovery, Qualitative, Lived experience

## Abstract

**Background:**

Peer support is a recovery oriented approach where consumers and carers are introduced to people with lived experience of the disorder who have recovered. Paid roles within health services for such consumer peer workers and carer peer workers (or ‘specialists’) are increasingly common. To date specific studies on such peer support for consumers with borderline personality disorder (BPD) and their carers has not been conducted.

**Methods:**

This qualitative study used interviews to explore perceptions and models of peer support for BPD from the perspectives of 12 consumers, 12 carers, and 12 mental health professionals. Participant responses were analyzed using reflexive thematic analysis within a phenomenological methodology.

**Results:**

All groups described how consumer peer workers may provide hope, connection, and validation to a consumer’s lived experience. Offering both traditional mental health treatment plus peer support, and giving consumers choice regarding a consumer peer worker was welcomed. Differences in opinion were found regarding the consumer peer worker’s role in relation to the mental health team, including whether consumer peer workers should access medical records. Perspectives differed regarding the consumer peer worker and carer peer worker positions, highlighting potential role confusion. Carers discussed the value of receiving support from carer peer workers and consumer peer workers. Mental health professionals described how consumer peer workers can experience workplace stigma and problems with boundary setting, and acknowledged a need for peer workers to be valued by having a duty of care and confidentiality code to follow and be offered supervision.

**Conclusions:**

Two models of peer support for BPD emerged: an integrated model where consumer peer workers work within the mental health team, and a complementary model where consumer peer workers are separate from the mental health team. Based on these findings we provide recommendations for services to help support such peer work for consumers with BPD and their carers.

## Background

Borderline personality disorder (BPD) is a severe mental condition that is characterized by disturbances in emotion regulation, identity, relationships with self and others, and impulsivity [1]. The disorder is prevalent in approximately 1 to 2% of the general population, 10% of mental health outpatients, and 20% of psychiatric inpatients [1, 2]. Psychological therapies for the treatment of BPD show variable treatment outcomes, including small to large effects in reducing symptomatology [3, 4]. Treatment outcomes may depend on a variety of factors including BPD severity, treatment length and treatment type. From a consumer perspective, recovery from BPD may involve symptom reduction, but also the generation of hope and participation in meaningful activities and relationships [5, 6]. Services for consumers with BPD which focus on recovery oriented outcomes have been recommended [5, 7]. In addition, carers who support consumers with BPD often experience high levels of stress and grief [8], and receive limited support for themselves [9]. Peer support may be beneficial for consumers with BPD and their carers to help enhance recovery. Peer support has been recommended for consumers with BPD to improve relationships and sense of self [10], and to improve personality disorder services [11, 12].

Peer support is acknowledged internationally as part of a recovery oriented service [13]. Peer support occurs when a person with a lived experience of mental illness provides support to another person with a mental illness [14]. Consumer peer workers are consumers with a lived experience of mental health problems, and carer peer workers are carers with a lived experience of supporting someone with mental health problems. Consumer peer workers and carer peer workers provide peer support services, whereas consumers and carers may or may not be in a peer worker role. Consumer peer workers and carer peer workers may have a role within mental health services or peer-run organizations, or may work independently [14]. The consumer peer worker and carer peer worker role varies across different services [15, 16]. In many cases, consumer peer workers and carer peer workers require training before starting the role and ongoing training and supervision is provided within the role, although the role may be provided based only on the peer worker’s shared experiences with consumers or carers [16, 17]. Due to the diverse roles of consumer peer workers and carer peer workers, consumers and carers can be supported in many ways, including peer workers providing informational, emotional, and social support [15]. For instance, a consumer peer worker may provide support to a consumer by facilitating a group which provides skills and resources [18], or they may provide support by listening to a consumer’s experience through a lens of shared understanding [19]. Administrative and clinical tasks, such as note writing and risk assessment, are sometimes completed by consumer peer workers, although different perspectives exist as to how and if those tasks should be part of the consumer peer worker role [15, 16, 20]. In addition, consumer peer workers may perform roles similar to mental health professionals, such case management, or have a separate role, such as advocacy [21]. Various models have been proposed which suggest ways of delivering peer support [22], such as peer supported Open Dialogue [23]. With any peer support role or model, experiential knowledge of consumer peer workers can be used to build rapport with consumers, and this characteristic differentiates consumer peer workers from mental health professionals [24]. The unique aspects of peer support may be maintained by peer support principles, including supporting safe relationships founded on shared lived experience, mutuality and reciprocity, promoting experiential knowledge, giving choice and control to peer workers regarding the way that support is provided and received, and empowering peer workers to use their strengths and find support through other peer workers and the community [13, 25]. For the purposes of this paper and its exploratory aims, peer support or support will be broadly defined as any form of assistance or encouragement provided to consumers or carers by consumer peer workers or carer peer workers.

Qualitative data indicates that peer support services can be beneficial for consumers and carers. Consumers often report feeling understood, hopeful, and more comfortable when conversing with a consumer peer worker [26–28]. One previous qualitative study analyzed audio recordings of a self-help group for consumers with BPD facilitated by a consumer peer worker and a mental health professional [29]. Results showed that humor, receiving praise and hearing the shared experiences from other group members and the consumer peer worker were helpful for consumers with BPD. Carers who support consumers with mental health problems describe valuing emotional and informational support from carer peer workers [30]. Consumer peer workers also express benefitting from providing peer support, including enhanced self-awareness and self-esteem [31–34]. In addition, mental health professionals describe how consumer peer workers provide organizations with a unique expertise of their journey through the mental health system which can add credibility to services [28, 35].

Quantitative evidence, although limited, has suggested that consumer outcomes may not differ when the same service is provided by consumer peer workers or mental health professionals [21, 36]. Mixed evidence exists for consumer outcomes when peer support is provided as an adjunct to traditional services [36], and change mechanisms regarding peer interventions are unclear [37]. Peer support provided to carers and families in a group setting also shows improved outcomes for carers, including greater empowerment and lower anxiety [38]. Although some positive outcomes exist for peer support for consumers with severe mental illnesses and their carers, further evidence is needed to understand and support peer delivered interventions [39]. In addition, peer support research often excludes individuals with BPD and does not clearly define the consumer peer worker role or the model of peer support used [21, 36]. Models of peer support for individuals with BPD do not exist in the literature, and models developed for other mental health problems may not be suitable for consumers with BPD due to the unique experiences of individuals with BPD [40].

Considering the perspectives of consumers and carers may be important to improve services for individuals with BPD [41]. A systematic review of 38 studies on consumer and carer perspectives of services for individuals with BPD found that consumers and carers had different foci, including carers not receiving appropriate recognition from health professionals, and consumers describing the importance of their relationship with a health professional [40]. Exploring health professional perspectives may also be important because professional perspectives can differ from those of consumers with mental health problems and carers [42, 43]. Multiple studies have investigated perspectives regarding peer support for mental health problems, and consumers and professionals have expressed different views regarding the consumer peer worker role description [16, 17]. Therefore, considering the perspectives of consumers, carers, and professionals may be important due to potential differences in opinion, and could be particularly valuable regarding enhancing services for individuals with BPD.

Despite some evidence that peer support for BPD may help improve recovery oriented outcomes for consumers with BPD and their carers, there are currently no specific studies exploring perceptions or models of peer support for individuals with BPD and their carers. The present qualitative study explored perceptions of peer support for BPD through the perspectives of consumers, carers, and mental health professionals. The aim of the study was to determine possible models and recommendations of peer support for BPD.

## Methods

### Participants

Participants were recruited using purposive sampling. First, national personality disorder groups were approached to identify leaders in peer work. Through a snowball recruitment strategy, individuals from services and locations which provide peer support to consumers with BPD or their carers were invited to participate, and participants were asked to identify other potential participants. Potential participants were informed of the purpose of the research and invited to participate through email. Ethics approval was provided by an institutional review board, and written informed consent was obtained from all participants. Participants included 12 consumers with BPD, 12 carers supporting someone with BPD, and 12 mental health professionals who had experience working with consumers with BPD and consumer peer workers or carer peer workers. Seven of the consumer participants were consumer peer workers, and 6 of the carer participants were carer peer workers. In the presentation of the data through quotations, consumers and carers who are consumer peer workers or carer peer workers will be distinguished from consumers and carers who are not peer workers. The sample allowed different perspectives from lived and professional experiences to be collected. Twelve individuals were included in each participant category, as this has been recommended in previous qualitative interview research to maximize the likelihood of saturation of themes [44]. Table 1 outlines the characteristics of the participants.

**Table 1:** Characteristics of Participants (*N* = 36)

*Type of Participant*	*Characteristics*	*n (%)*
Consumer (*n* = 5)	Female	5 (100)
Consumer peer worker (*n* = 7)	Female	7 (100)
Carer (*n* = 6)	Female	4 (66.7)
	Relationship to consumer	
	Parent	3 (50)
	Spouse/Partner	3 (50)
Carer peer worker (*n* = 6)	Female	6 (100)
	Relationship to consumer	
	Parent	5 (83.3)
	Spouse/Partner	1 (16.7)
Mental Health Professional (*n* = 12)	Female	7 (58.3)
	Occupation	
	Nurse	3 (25)
	Occupational Therapist	1 (8.3)
	Psychiatrist	1 (8.3)
	Psychologist	5 (41.7)
	Social Worker	2 (16.7)

### Procedure

Semi-structured interviews were conducted with individual participants. Interviews were facilitated by the same interviewer either over the phone or in-person, depending on the preference of the participant. Interview questions were based on a guide that was created by the authors. Open-ended questions were administered to all participants, with slight variations depending on whether they were a consumer, carer, or mental health professional. Consumers and carers were asked to describe their lived experience of BPD and peer support. Mental health professionals were asked to describe their role and their experience with BPD and peer support. The remainder of the questions were the same for consumers, carers, and mental health professionals. Participants were asked what role they wanted a peer worker to take, what characteristics they wanted the peer worker to have, and how the contributions of peer workers and mental health professionals are different or similar. Questions that were asked included, “What contributions would a peer bring to individuals with BPD, compared to mental health clinicians?” and “What would you want the role of a peer support worker to be for individuals with BPD?” Appropriate follow-up questions were asked as required. The interviews were audio recorded and transcribed verbatim. Information that was personally identifying was removed from the transcripts. Interviews ranged from 26.4 to 77.6 min, averaging 48.2 min. Consumer and carer participants were compensated for their time.

### Data analysis

Data were analyzed to understand participants’ lived experiences using an interpretive phenomenological methodology to ensure preconceptions were ‘bracketed’ [45]. Analysis of the data was performed using ‘reflexive thematic analysis’ as described by Braun, Clarke, Hayfield, and Terry [46]. By repeatedly listening to audio recordings and reading transcripts, a thorough understanding of the data was obtained. Using NVivo 12, participant statements were coded into nodes with similar meanings using an inductive orientation. Next, themes were constructed by combining nodes with conceptual likeness, and themes were revised as necessary to reflect the data. Coding was performed independently. Throughout the coding process, several discussions within the research team ensured that the themes depicted participant responses. A second researcher coded four interview transcripts to also determine inter-rater reliability. The amount of agreement between the two researchers was calculated using Cohen’s kappa coefficient. The inter-rater reliability was κ = 0.71, indicating a moderately high level of agreement [47]. Themes were then presented to participants to validate themes derived and to ensure that accurate meaning was captured. Final themes were then agreed on as presented here.

## Results

### Shared perspectives of consumers, carers, and mental health professionals

There were themes that all three groups endorsed and are described below.

#### Consumer peer workers provide shared experiences to consumers that bring hope and connection

Consumer peer workers conveying hope to consumers, including demonstration that recovery is achievable, was strongly recognized by consumers, carers, and mental health professionals. “*It really does feel like you’re never going to get better when you are unwell, and unfortunately, I think a lot of clinicians still hold quite a stigma regarding BPD that um, clients with that diagnosis won’t recover and won’t lead meaningful lives, so I think it’s, um, really helpful to have someone with that lived experience in that role and doing quite well, um for the clients to be able to see that recovery is actually quite possible, um and you can lead a meaningful life*” (Consumer Peer Worker 1).

A peer support relationship was often described as casual, including the use of everyday conversations and informal language, which allows consumers to feel comfortable. “*They (consumers) understand what you mean when you say, ‘I waited 12 hours.’ They’ve waited 12 hours. So, there’s a way, it’s kind of like a short-hand, I guess, and language you speak to someone with a lived experience, which means that you don’t have to be so descriptive*” (Consumer Peer Worker 3). In contrast to limited disclosure from mental health professionals, consumer peer workers are open in sharing their thoughts and experiences, which can help other consumers open up through quick connection and trust. *“I (consumer peer worker) was telling her (consumer) about when I get stress sometimes I get this thing where I’ve got to repeat these phrases over and over in my head [laughs]. And then she started to let me know about something she’d been saying since 2009 in her head”* (Consumer Peer Worker 4). Reciprocal relationships with consumer peer workers were compared to existing power imbalances with mental health professionals. “*There’s a reciprocal respect that comes from that (peer support) where there’s not a power differential that I think is often existing in the clinical relationship*” (Mental Health Professional 4). Several participants differentiated consumer peer workers from mental health professionals with a lived experience of mental health problems because professionals don’t explicitly “*use that (their lived experience) as their tool of engagement*” (Mental Health Professional 4).

#### Consumer peer workers understand and validate consumer experiences

The majority of consumers, carers, and mental health professionals described consumer peer workers understanding and validating consumer experiences. Mutual understanding can provide consumers with validation and comfort, which may differ to experiences with mental health professionals. *“I guess there is a difference between a psychologist being able to say, ‘uh, that must be really difficult for you’ and someone else (a consumer peer worker) being able to say ‘I, I remember how difficult that was’”* (Consumer 6). Many consumers described stigmatizing responses they had received from other people, services, and mental health professionals. Consumers, carers, and mental health professionals reported that consumer peer workers can validate the unique experiences of BPD which are often not understood by others, helping consumers feel less judged and isolated. “*They (consumers) don’t feel alone, they don’t feel weird or different. Last week one of the girls said she doesn’t feel like she’s got two heads. She feels like she’s a normal kind of person and that she’s not being judged*” (Consumer Peer Worker 5).

#### Consumer peer workers provide support to consumers as they transition from acute to community care

Consumers, carers, and mental health professionals described how consumer peer workers provide input and support as a consumer moves from acute crisis, inpatient stays, to community care. Many participants described how consumer peer workers advocate for consumers when treatment decisions are being made. *“Within like hospital settings and that sort of thing um, clients feel like they’re not being heard by clinicians, um so it might be, um, being pushed for discharge before they are ready, or it might be being pushed into a type of therapy that they don’t want to do... so having a peer recovery worker who understands that experience and is able to advocate for the rights of the client is really important*” (Consumer Peer Worker 1).

In addition, consumer peer workers can provide emotional support to consumers during a crisis, inpatient stay, or hospital discharge. *“If they (consumers) come onto the ward they’d actually ask for me … and I’ll just sit down and just listen to see where they’re at”* (Consumer Peer Worker 2). Many participants suggested that consumer peer workers can spend time with consumers in the community, including meeting at cafes or attending appointments, which allows consumers to feel empowered and develop interpersonal skills. However, one consumer peer worker believed consumer peer workers should not engage in community activities because it is *“not unique to their lived experience”* (Consumer Peer Worker 4).

Many participants suggested that consumer peer workers share and role model their personal use of skills and strategies which may be relatable and hold more authority, compared to skills shared by mental health professionals. *“They (consumers) respond differently, as to, like one of the other clinicians, teaches them about a skill as to when I bring up my lived experience and say when I felt like that I, I found this really useful... you can tell that it’s quite meaningful for them to have that experience”* (Consumer Peer Worker 1). According to many participants, consumer peer workers can connect consumers to resources, help them navigate the mental health system, and provide education about BPD. Most participants described how consumer peer workers can facilitate Dialectical Behavior Therapy (DBT) or support groups. About half of the participants suggested that consumer peer workers can help consumers with homework and skills taught in therapy. *“If they’re (the consumer) doing DBT, and they’ve got exercises to do, and they don’t know how to do them, and that (consumer) peer worker has done DBT, and understands it, yeah, to have somebody maybe help you through your exercises”* (Carer Peer Worker 3).

#### All groups agreed that it is important to allow consumer choice in selecting a peer worker in which to work

Consumers, carers, and mental health professionals suggested providing choice to consumers regarding their consumer peer worker. All participants identified the importance of a suitable match between a consumer and consumer peer worker. “*It would be lovely if you could say to a consumer, ‘Would you like to work with a peer support worker, what gender, what age?’ I mean, because you know, it’s I suppose trying to match it as much as possible*” (Mental Health Professional 8).

The majority of consumers and carers described how a consumer peer worker with a diagnosis of BPD was beneficial. *“I, personally, would prefer someone (a consumer peer worker) with BPD. Um [pause], not another diagnosis … a lot of my own experience with BPD could only really be understood by somebody else with BPD”* (Consumer 5). Other participants described how a consumer peer worker should have similar experiences as consumers and an understanding of BPD, but not necessarily a BPD diagnosis. “*If you’ve experienced extreme distress, you’ve experienced mistreatment in the public mental health system … those things are still quite important for the client to know that you’ve experienced but it doesn’t necessarily need to come with a diagnosis of BPD*” (Consumer Peer Worker 1). A consumer peer worker’s identified gender, age, recovery stage, culture, linguistic background, sexual orientation, and personality were important considerations for some participants. For example, consumers might feel more comfortable with a same gendered consumer peer worker. Many participants described offering choice to consumers regarding their preference of individual or group support, whether to access peer support, and how often support is provided. Several participants described how consumers may feel more comfortable engaging with consumer peer workers compared to other services or treatments.

#### All groups agreed that it is important to consider offering support for consumers from both mental health professionals and consumer peer workers

The majority of consumers, carers, and mental health professionals described the importance of consumers receiving support from consumer peer workers and mental health professionals, due to their unique contributions. Having both types of support available to consumers can maximize benefits for consumers. “*If the debate becomes peer work versus something else, we’ve actually lost the consumer in that conversation. So, I think peer support has a really important role in all types of mental health support and treatment but so, too, has good clinical care as well*” (Mental Health Professional 2). Therefore, increasing the amount of consumer peer workers and accessibility of peer support services was recommended.

#### Areas of disagreement

There were some areas that consumers, carers, and mental health professionals held different views about various roles and functions of the consumer peer worker and carer peer worker. For example, there were differences in opinion about how integrated the consumer peer worker should be in a clinical mental health team.

Some consumers advocated for consumer peer workers being considered a part of the mental health team, including writing notes and accessing medical records, as this was seen as helping to validate and integrate them as part of the team. Many consumers described how information in medical records helps consumer peer workers understand them, which can help them feel cared for. “*I can speak to someone who has an understanding of what it is like and can also see sort of my past up to this point. That’s going to probably immediately make me feel more comfortable and like also like they care more and have put in the time*” (Consumer 6). Consumer peer workers and mental health professionals can exchange information about consumers by writing their observations in medical records and through verbal communication. Shared information between consumer peer workers and mental health professionals can keep consumer peer workers and consumers safe by increasing awareness of risk concerns, allowing continuity of care, and improving interactions with consumers. *“It is important to have (consumer) peer workers writing clinical notes and being able to view clinical files so it helps them work with clients in the best possible way, and it makes the (consumer) peer worker’s … opinions and their engagement with clients known to other clinicians in the team”* (Consumer Peer Worker 1). Some consumers discussed the importance of providing note writing training and ensuring time spent note writing does not take away from spending time with consumers. Several consumers believed that consumer peer workers writing notes or accessing medical records would not negatively influence the relationship with consumers. Trust can be fostered with consumers if the note writing process is made transparent, and the content of the notes is collaboratively chosen between consumers and consumer peer workers. *“It’s down to the person who is being supported … what information do they want disclosed? And – and they may not even want that to happen”* (Consumer Peer Worker 6).

In contrast, some consumers considered that it is important to separate the consumer peer worker role from mental health workers, and thus were not willing for consumer peer workers to access medical reports or write notes into hospital files. For example, two consumer peer workers described how consumer peer workers should remain separate from the mental health team and not write notes or access consumer medical records. They reported how the consumer peer worker role is different from the role of mental health professionals, and requiring them to do the same tasks takes away from the unique reciprocal role and expertise of the consumer peer worker. In addition, medical records may not accurately represent consumers, and consumers may be less open with consumer peer workers who have read their medical record. *“[Consumers are] less likely to engage with you (consumer peer worker). Um if – if you’re doing that, if – if you’re reading notes, writing notes. So they’re not going to be open with you”* (Consumer Peer Worker 2). Both consumer peer workers emphasized how all information a consumer peer worker learns about a consumer should come through conversations with the consumer. “*Often what’s actually written in those records really doesn’t match what it is the person’s saying. And that relationship is the most important aspect of peer work, therefore, you know, as a (consumer) peer worker I would want the person to be telling me their story themselves”* (Consumer Peer Worker 7).

Many carers described the value of integrating consumer peer workers into the mental health team, although some were cautious of doing so. Carers suggested that when consumer peer workers view medical records it can help them understand consumers and aid with continuity of care. *“If the (consumer) peer worker had some access to notes to know what had gone before. You know, what sort of things might upset them (the consumer), what sort of things they enjoyed; all that sort of stuff”* (Carer Peer Worker 4). By reading medical records, consumer peer workers can learn about risk concerns, potential triggers for consumers, and whether consumers are engaged with other services. Several carers identified the importance of training consumer peer workers and carer peer workers in note writing, confidentiality, and duty of care issues. Some carers expressed the importance of consumers consenting to consumer peer worker’s writing notes, and communicating with consumers about what information will be included in the notes. Several carers believed that consumer peer workers accessing medical records would benefit their relationships with consumers because consumer peer workers are non-judgmental and have built trust with consumers. Carers acknowledged that information that is accessed from medical records should be complemented by listening to the experiences of the consumers. *“I think it’s important that they (consumer peer workers) know a lot about the person that they are supporting. But I think a lot of that should come from the person as well”* (Carer Peer Worker 1). In contrast, some carers were wary about consumer peer workers reading consumer medical records due to the negative consequences for the peer support relationship. *“It becomes like a session. It definitely changes the whole dynamic”* (Carer 2).

In addition, some mental health professionals identified the value of having consumer peer workers being included as part of the team, whereas others saw value in there being a separation of role and function. Mental health professionals described how verbal communication and note sharing between consumer peer workers and mental health professionals can help professionals understand and support consumers. *“There’s actually no real reason why they (consumer peer workers) shouldn’t be contributing to the notes … What it did is create more information that the whole team had to support a person, as opposed to a sort of two-tier system where the peers worked over here, and the clinicians worked over there”* (Mental Health Professional 2). Communicating with consumers about note writing and the consumer peer worker’s involvement in the mental health team was recommended by several professionals. The ability to access the notes of consumer peer workers can also help professionals understand the consumer peer worker role and what they are doing with consumers, and vice versa. *“If they (consumer peer workers) don’t know what the rest of the team they’re working with is doing, and if the rest of the team can’t know what they’re doing, all we’ve created is risk and confusion”* (Mental Health Professional 11). Clarifying the responsibilities of consumer peer workers and mental health professionals regarding documentation of consumer risk issues was suggested to protect consumers, consumer peer workers, and professionals. Many professionals described how including consumer peer workers in the mental health team, including the ability to access medical records and write notes, helps professionals acknowledge the value of consumer peer workers. *“We need to view peers as colleagues and as, um – you know, as I said, privilege their expertise and their knowledge as much as we privilege our own and so I think if we’re working alongside peers they should be entitled to, and able to, complete clinical tasks such as writing notes and, you know, viewing medical records”* (Mental Health Professional 7)*.* However, some professionals were hesitant about involving consumer peer workers in the mental health team, and acknowledged how a consumer peer worker’s access to medical records could negatively impact the peer worker’s relationship with consumers. *“[Reading clinical notes] would become a barrier to you (the consumer peer worker) actually sitting with a client and go, okay, what’s important to you today … and I think it might distract from being really, kind of, here and now”* (Mental Health Professional 9). Some professionals identified risk around consumer peer workers becoming unwell, such as being triggered by reading notes, and not maintaining appropriate boundaries or confidentiality. *“The risk is of course, that if people (consumer peer workers) become unwell that they may have issues around their own boundaries and maintaining confidentiality”* (Mental Health Professional 10).

Consumers, carers, and professionals also had different perspectives regarding whether carer peer workers should provide support to consumers, and consumer peer workers should provide support to carers. Several carers believed that support from carer peer workers would benefit consumers, whereas other participants expressed that consumers would be better supported by consumer peer workers. *“I think consumers often relate better to consumer peer workers than they do to carer peer workers”* (Mental Health Professional 2). Most participants thought that consumer peer workers could support carers, although some consumers believed that consumer peer workers should predominately support consumers. Two consumer peer workers suggested having distinct roles for consumer peer workers and carer peer workers to avoid role confusion. *“Do you use a, um, (consumer) peer support worker or a carer peer support worker, and that’s really a blur because what we do here is I don’t deal with anybody’s carers … I guess my thought is it’s going to be hard for people to work across more than one space”* (Consumer Peer Worker 4).

### Other perspectives

There were several themes that were constructed based on the unique perspectives of consumers, carers, and mental health professionals that are described in the next section.

#### Consumers identified the importance of consumer peer workers maintaining well-being and being valued and supervised in their workplace

Consumers described how consumer peer workers can often balance organizational policy or the opinions of mental health professionals and the best interest of the consumer, which can leave them with little power. *“If you’ve got, yeah, the (consumer) peer worker on the side of the consumer but you’ve got the rest of the team on another, you know, the (consumer) peer worker’s going to become unwell and it’s not, not a good model”* (Consumer Peer Worker 4). Several consumers reported that consumer peer workers are often poorly treated and stigmatized by organizations, mental health professionals, and peer workers with other diagnoses. *“The amount of bullying and stigmatizing that goes on within the peer support workers, it’s horrible”* (Consumer Peer Worker 3).

Consumers described the importance of consumer peer workers maintaining their own well-being and being valued by mental health professionals and organizations. Self-care can be strengthened by setting personal boundaries regarding what information they disclose to consumers and appropriately managing time spent with consumers. Some consumer peer workers described how interacting with consumers in itself was helpful to them. *“To give back is just, um, is so satisfying”* (Consumer Peer Worker 6). Consumers described the importance of supervision being provided to consumer peer workers by mental health professionals to help them maintain self-care. Several consumers suggested hiring consumer peer workers who are far enough in their recovery, including the ability to set appropriate boundaries and articulate a story of recovery. “*At some point I think people (consumers) feel that they’re ready to become a (consumer) peer worker but the story they tell is very much an illness story, and so, ‘This is – this is what’s happened to me, this is my experience,’ rather than, you know, ‘These are the things that I did to recover, these were the things that have helped me to recover*’” (Consumer Peer Worker 7). Allowing choices within the consumer peer worker role and increasing compensation and work opportunities for consumer peer workers were also recommended.

#### Carers emphasized how carer peer workers have an important role in providing emotional and informational support to carers

All carers described how peer support could be provided to carers by carer peer workers. Peer support provided by carer peer workers to carers can help carers support consumers. *“If you can support the carer, they’re going to be a lot better at supporting the consumer”* (Carer Peer Worker 4). Carer peer workers can offer hope to carers by sharing how they have made it through difficult times. *“I’ve had people in our support group that are loved ones that say, well, we were told by, you know, the psychologist or the psychiatrist that, you know, our loved one would never get well, they’d never have a job. They’d always be like this, it would always be difficult. And that’s what they believed. Until all of a sudden, they meet someone who’s not only saying there’s hope and there’s ways to get well, they’re saying there’s hope, there’s ways to get well and we know this as fact”* (Carer Peer Worker 2). Carers described being validated and comforted by carer peer workers who can understand their unique experiences, unlike other people who may judge them for the way they interact with the consumer they support. *“A (carer) peer (worker) would understand why you’re nervously responding to your phone all the time and midway through a conversation, you’d be texting, and trying to have a cup of coffee with a friend, but, yeah, a peer wouldn’t judge you for that”* (Carer Peer Worker 3). One carer described how she would prefer support from a carer peer worker who supported someone with BPD rather than another mental health problem, due to the shared understanding.

Many carers described how carer peer workers can advocate for their needs, and provide information about BPD, resources, and the mental health system. *“With the resources and information that I’ve collected over the years, I’ve been able to pass that on and offer support, um, to encourage them (carers) to attend workshops and basically get any information that they can about BPD, so that they are prepared, um, and that they know how to handle – often handle situations and behaviours, um, that the person they care for presents with”* (Carer Peer Worker 5). Carer peer workers can provide suggestions to carers about effective ways to communicate with the person they support, and provide practical advice. Group meetings may be particularly important for carers to allow them to hear multiple perspectives of how to potentially handle a situation. In addition, group meetings for carers could be tailored to the needs of siblings, children, and partners. Carer peer workers helping carers practice self-care and providing emotional support to carers, including allowing them to share and listening to them, was important for many carers. *“It takes a big load off, um, a lot of carers shoulders because it’s just more being able to vent, um without being judged”* (Carer Peer Worker 4). Several carers recommended improving the accessibility of peer support for carers, including having carer peer workers that can be contacted during a crisis. One carer peer worker recommended that carers receive support from carer peer workers who have more years of experience being a carer. *“Having these experienced carers – um, when I say, experienced, it’s more, they are in a good place and they’ve been in a carer role for quite some time … they can come in and – and help share the good – the good stories as well”* (Carer Peer Worker 1).

#### Carers identified the value of hearing and learning from the experiences of consumer peer workers

The majority of carers described how they could be supported by consumer peer workers. Consumer peer workers can tell carers how they felt when they were in the consumer’s situation or share what consumers are learning in therapy to help carers understand what consumers are experiencing and how they can best support them. *“Being involved with other consumers who have BPD as they have shared what’s helped them, how they’ve coped, what’s been detrimental and hasn’t helped has given me a lot of insight and awareness. So I’ve been far more informed in the way I handle things with my daughter”* (Carer Peer Worker 6).

#### Carers identified the value of having access to respite, and the possible role of consumer peer workers in providing that support

Carers can receive respite when consumer peer workers spend time with consumers in the community, including helping consumers with education and employment goals. *“Most carers of the – would just like to find something that would make their children less isolated, and also give the carer a – a – a break. A bit of respite for a carer, really, if they go off with the peer support worker, even if it’s for an hour. Um, that gives the carer that hour off”* (Carer Peer Worker 4). However, several carers described negative experiences of the person they support being influenced by a consumer with BPD who was not in recovery, and recommended that consumer peer workers are further along in recovery compared to the consumers they support.

#### Mental health professionals identified how consumer peer workers and carer peer workers inform and improve mental health care

According to mental health professionals, consumer peer workers and carer peer workers can help change services to become more recovery oriented. Several professionals described how consumer peer workers helped them interact with consumers more effectively and compassionately. *“When you have peer support workers, for any diagnosis, but particularly for, for personality disorders, you get less incidents of one to one, especially in seclusion, and over medication and all the usual punitive things mental health services tend to do to what they perceive as difficult people”* (Mental Health Professional 2). Interacting with consumer peer workers and carer peer workers can help mental health professionals be mindful of the language they use, and be educative about alternative appropriate language. *“I think there can be sort of peer consultation for professionals, you know, which would, I think, also help us manage our language and the way that we speak about people, um, because sometimes I think we can – professionals can use short cuts and can inadvertently use language that is unhelpful or, um, inadvertently blaming of people... they (consumer peer workers and carer peer workers) might help us, um, understand the impact of language”* (Mental Health Professional 7). In addition, the ways that consumer peer workers write notes may be more recovery oriented and inform the note writing of mental health professionals. Further, including consumer peer workers in interventions delivered by professionals can help professionals support consumers. *“I mean it would be great if they (consumer peer workers) could share their story as part of the, sort of, therapeutic intervention that we have”* (Mental Health Professional 10).

#### Mental health professionals described the value of consumer peer workers and looked for opportunities to strengthen their contribution

Mental health professionals acknowledged how consumer peer workers have been mistreated and undervalued by services and mental health professionals, including hesitation by some workplaces to hire consumers with BPD. *“There’s probably stigma from employers about the idea of hiring people who, um, whose lived experience is of BPD”* (Mental Health Professional 4). Professionals described how clarifying the consumer peer worker’s purpose in an organization can help them understand and value the consumer peer worker role. Several professionals were concerned about the boundaries between consumer peer workers and consumers, such as the relationship being too casual, particularly because consumers with BPD may have difficulties understanding appropriate boundaries. Consumer peer workers setting boundaries around mobile phone contact and community contact with consumers was suggested to help prevent potential risk issues. *“We kind of established with the voluntary peer support worker that, you know, the contact is just to be had, you know, at the nominated times in the building here or the park nearby so that it, I suppose as much as a kind of protective mechanism for her, um, as also for my consumer”* (Mental Health Professional 8). Mental health professionals described the importance of professionals providing valuable information to consumer peer workers through supervision and training. *“The clinician can provide some, some support to the (consumer) peer worker about, you know, um, yeah, anything that the clinician has knowledge about that the (consumer) peer worker might not”* (Mental Health Professional 5).

## Discussion

This novel study used individual interviews to explore perceptions of peer support and peer support models for individuals with BPD and their carers from the perspectives of consumers, carers, and mental health professionals. Findings highlight the importance and value of providing peer support to consumers with BPD and their carers, and the need for consumer peer workers to be valued by mental health professionals and organizations. In addition, contrasting views regarding roles and functions of consumer peer workers within the mental health team were found.

Results showed that the shared lived experiences of consumer peer workers were described by participants as contributing to recovery by providing hope, connection, and validation to consumers with BPD, which is consistent with previous qualitative research on peer support for mental health problems [26, 27]. Given the stigma and unique experiences related to BPD [48], peer support might be particularly valuable to help consumers feel understood and validated [29]. Further, consumer peer workers were valued for helping consumers with BPD as they move from acute to community care, as well as providing support to carers. For example, participants described how consumer peer workers can provide skills to consumers based on their own experiences, which is consistent with previous recommendations that psychological education for BPD can be provided through peer support [10]. The findings suggest it is important for consumer peer workers to discuss their role with consumers, including what types of peer support are available and whether the consumer peer worker has consent to interact with carers [49]. Carer peer workers can also support carers, which may help improve their well-being and help them support consumers.

Participants had different opinions regarding consumer peer workers supporting carers and carer peer workers supporting consumers. To prevent role confusion, organizations and peer support guidelines may need to clarify whether consumer peer workers and carer peer workers provide support to both consumers and carers, or whether the roles are distinct in that consumer peer workers provide support to consumers and carer peer workers provide support to carers. Providing choice to consumer peer workers and carer peer workers regarding their role description may also be important [25].

The shared view of consumers, carers, and mental health professionals regarding the importance of having a suitable match between consumers with BPD and consumer peer workers, including opportunities for consumers to choose their consumer peer worker, was consistent with research that shows that peer support is based on shared personal experiences [13, 24]. Further, providing choice to consumers regarding who they enter a peer support relationship with upholds peer support principles [13, 25, 50]. Therefore, organizations may consider hiring multiple consumer peer workers to ensure consumers have options when choosing a consumer peer worker. In addition, the perspective of participants that consumer peer workers and mental health professionals are both necessary to provide optimal treatment demonstrates how both have unique knowledge and skills that can help consumers with BPD [24]. Consulting with consumers regarding the support they receive, and offering to include peer support in treatment plans is recommended [42]. Further, increasing the availability and accessibility of peer support for both consumers with BPD and their carers is important, including making health professionals aware of peer support options to inform referrals [51].

In line with previous research, consumers and mental health professionals described how consumer peer workers can be mistreated and unsupported by organizations [52], highlighting the need for consumer peer workers to be valued and offered supervision and training [20, 53, 54]. When a consumer peer worker’s purpose is clear to the mental health team, it may enable mental health professionals to better value and work effectively with consumer peer workers [17]. As in prior research, mental health professionals expressed concerns regarding boundaries between consumer peer workers and consumers and the potential negative impact on consumer peer workers [55]. Mental health professionals and carers also indicated that consumer peer workers and carer peer workers may have difficulty with confidentiality, confirming previous research which indicates that consumer peer workers may not want to break confidentiality in risk situations due to their relationship with the consumer [20]. Duty of care obligations for consumer peer workers and carer peer workers may differ across services and organizations, highlighting a need to ensure that consumers and carers are protected when peer support services are delivered [20]. The finding that consumer peer workers and carer peer workers inform and improve the practices of mental health professionals is also consistent with previous research [56, 57]. Therefore, consumer peer workers, carer peer workers, and mental health professionals providing consultation to one another is important and could be further facilitated by organizations.

The present findings suggest that consumers with BPD and their carers experience validation from consumer peer workers and carer peer workers which may combat the stigma toward BPD. Consumer peer workers and carer peer workers may also alter the practices of mental health professionals to be more recovery oriented. Providing peer support in services where stigmatized responses are heightened, including non-specialist BPD settings such as the emergency department [12], may be beneficial for consumer and carers, and could help alter the practices of professionals. However, the current study also found that consumer peer workers with BPD can experience stigma from other consumer peer workers and mental health professionals, which is consistent with previous research [52]. Therefore, assessing an organization’s workplace stigma toward BPD and changing if indicated may be essential prior to the introduction of consumer peer workers and carer peer workers.

### Two models of peer support for BPD

Participants expressed mixed views regarding consumer peer worker involvement with medical records and notes shared by the mental health team. While many consumers and carers described how consumer peer workers reading medical records positively influences interactions with consumers, others described how reading medical records negatively influences interactions and detracts from the relational aspect of peer support. In addition, mental health professionals described how they are better able to understand the role of consumer peer workers when consumer peer workers access medical records and write notes. Overall, there was a preponderance of participant opinions favoring inclusion of consumer peer workers in the mental health team. Previous research indicates that consumer peer workers can share consumer information with other mental health team members without negatively influencing the peer support relationship [20], particularly when consumer peer worker notes are written in a recovery oriented, non-judgmental manner [58]. However, sharing information about consumers with mental health professionals can be difficult for consumer peer workers [20], and until the present study there was an absence of research on how consumers view this topic.

Therefore, the current study proposes that consumer peer workers can have an integrated or complementary role in relation to the mental health team. Table 2 summarizes these two models of peer support for BPD that were discussed by participants. This model is consistent with previous research which suggests that consumer peer workers can have an integrated or complementary role in mental health services [21, 59]. Organizations that adhere to either of these peer support models may benefit by providing consumer peer workers, other staff, consumers, and carers clarity regarding the consumer peer worker role. Previous research has acknowledged the importance of organizations clearly defining the consumer peer worker role and distinguishing the role from non-peer roles [56, 60]. Role clarity within the mental health team may help consumer peer workers feel more valued and understood by non-peer staff [16], potentially reducing mistreatment and stigmatization of consumer peer workers [61]. In the absence of a defined model of peer support in an organization, the shared lived experience that consumer peer workers offer may not be utilized, undermining the purpose of peer support [13, 62]. Therefore, when implementing either model, it is important for organizations to be guided by peer support principles [13, 25]. In addition, discussing the model with prospective and current consumer peer workers at an organization may be important to determine whether the model corresponds with the consumer peer worker’s values regarding peer support [25]. It is also imperative that services evaluate the effectiveness of an integrated or complementary model, including evaluating consumer outcomes and the relationship between consumers and consumer peer workers before and after a model is introduced. Following these analyses, we then pulled together a set of statements and probes for services to consider in establishing a peer workforce for BPD. Table 3 outlines these recommendations for services to support peer work for consumers with BPD and their carers.

**Table 2:** Two models of peer support for borderline personality disorder

Model 1: Consumer peer workers integrated in the mental health team	Model 2: Consumer peer workers complementary to the mental health team
• Consumer peer workers write notes and the notes are shared with other mental health team members. • Consumer peer workers read consumer medical records and the notes of other mental health team members. • Formalized consultations may occur between consumer peer workers and the mental health team.	• Consumer peer workers may or may not write notes and notes are not shared with other mental health team members. • Consumer peer workers do not read consumer medical records or notes of other mental health team members. • Informal discussions may occur between consumer peer workers and the mental health team.

**Table 3:** Recommendations for services to support peer work for consumers with borderline personality disorder and their carers

1. Evaluate an organization’s workplace stigma toward borderline personality disorder and consider altering if required before the introduction of consumer peer workers and carer peer workers.	
2. Inform the development and delivery of peer support based on peer support principles and the values of consumer peer workers and carer peer workers. Ensure duty of care and confidentiality codes are in place to guide practice.	
3. Clarify the consumer peer worker and carer peer worker role in collaboration with the peer worker, including whether consumer peer workers support carers and carer peer workers support consumers. Discuss the consumer peer worker and carer peer worker role with members of the mental health team, consumers, and carers.	
4. Clarify whether a complementary or integrated model of peer support will be used. For either model, clarify the selection criteria for consumer peer workers and carer peer workers, and how consumer peer workers and carer peer workers will be trained and supervised.	
5. Consider hiring multiple consumer peer workers to provide consumers with options to select a consumer peer worker.	
6. Consider including peer support in treatment plans for consumers.	
7. Consider offering support to consumers from both consumer peer workers and mental health professionals.	
8. Consider increasing the accessibility of peer support, and make professionals aware of peer support options to inform referrals.	
9. Consider organizing consultation and supervision between consumer peer workers, carer peer workers, and mental health professionals.	

### Limitations and future research

This study used a purposive sampling technique, which may have produced a biased sample [63]. Although we looked for leaders in the field, we are unsure how representative the views of these participants are compared to other consumers, carers, and mental health professionals. In addition, the majority of participants were female, including all consumer participants. Notwithstanding these limitations, the sample was drawn from diverse backgrounds, and thus provided rich and compelling perspectives on peer support for BPD, including lived experience perspectives. The developed recommendations for services synthesized important considerations in regards to the role, tasks and support provided to consumer peer workers and carer peer workers. Future research evaluating peer support services for BPD may explore the two models of peer support for BPD, evaluate the implementation of the aforementioned recommendations, provide information on how peer support for BPD is related to recovery oriented outcomes, and investigate how peer support for BPD differs from support provided by mental health professionals [37, 64].

## Conclusion

This study explored models and perceptions of peer support for BPD through the perspectives of consumers, carers, and mental health professionals. Previous research has not specifically explored perceptions of peer support for BPD, and the current findings show how peer support may benefit consumers with BPD and their carers. In addition, the findings indicate the importance of workplaces valuing and providing supervision to consumer peer workers with a lived experience of BPD due to stigma toward BPD. It is recommended that organizations clarify whether the consumer peer worker has an integrated or complementary role in relation to the mental health team, in collaboration with the preferences of consumer peer workers.

## Data Availability

Data from the current study will not be made available, as participants did not consent for their transcripts to be publicly released. Extracts of participant responses have been made available within the manuscript.
